# Effectiveness of acupuncture in the treatment of chronic sciatica from herniated disks: a systematic review and meta-analysis

**DOI:** 10.3389/fmed.2026.1689124

**Published:** 2026-01-22

**Authors:** Zhen Qu, Jiang-yi Ju, Han Qin, Yun-xia Ding, Li-hua Peng

**Affiliations:** Deportment of Orthopedics, Bishan Hospital of Chongqing Medical University, Chongqing, China

**Keywords:** acupuncture therapy, chronic sciatica, disc herniation, meta-analysis, ODI (Oswestry Disability Index), VAS (visual analog scale) score

## Abstract

**Introduction:**

Chronic sciatica, frequently attributable to lumbar disc herniation, imposes substantial burdens on patients’ quality of life and healthcare systems. Conventional interventions, including pharmacotherapy and physical therapy, are widely employed yet constrained by limited efficacy, necessitating the exploration of complementary modalities such as acupuncture. This systematic review and meta-analysis synthesizes evidence from randomized controlled trials (RCTs) published over the past decade (2015.05 – 2025.05) to evaluate the efficacy of acupuncture in alleviating leg pain intensity and improving functional mobility outcomes among patients with chronic sciatica.

**Methods:**

We searched four databases (PubMed, EMBASE, Cochrane Library, Web of Science) for RCTs on acupuncture versus control (sham acupuncture, standard care, or conventional acupuncture). Studies included adults with sciatica from herniated disks, assessed via VAS for pain and ODI for function. Data were pooled using random-effects models with subgroup analyses for heterogeneity. Bias risk was assessed with the Cochrane tool.

**Results:**

Eleven RCTs (*n* = 868 participants, predominantly from China) were included. The results demonstrated that acupuncture significantly reduced leg pain VAS scores compared with those of controls (SMD = –1.08, 95% CI: –1.41 to –0.75), with consistent efficacy across the sham acupuncture (SMD = –1.05), standard care (SMD = –1.02), and conventional acupuncture (SMD = –1.12) subgroups. Acupuncture also improved functional disability (ODI: SMD = –0.57, 95% CI: –0.84 to –0.31; 7 studies, *n* = 621). The results indicate both statistical significance and clinical relevance, supporting acupuncture as an effective intervention.

**Discussion:**

Acupuncture effectively alleviates pain and aids functional recovery in chronic sciatica, highlighting its role in multidisciplinary pain management. However, methodological limitations in the extant literature necessitate cautious interpretation, and future high-quality RCTs are warranted to strengthen evidence-based clinical implementation.

**Systematic review registration:**

https://www.crd.york.ac.uk/PROSPERO/view/CRD420251067853, identifier (CRD420251067853).

## 1 Introduction

Chronic sciatica, a frequent complication of lumbar disc herniation, arises from the compression of nerve roots, leading to radiating pain along the sciatic nerve, which is often accompanied by lower limb numbness, muscle weakness, and dysfunction (1–3). The global lifetime incidence of sciatica ranges from approximately 1.6 to 43%, with approximately 10 to 40% of cases progressing to a chronic condition lasting more than 12 weeks (4, 5). While most patients find relief through conservative treatments, research indicates that approximately 45% do not experience significant symptom improvement within 1 year, and 34% may endure pain for more than 2 years, substantially diminishing their quality of life and increasing healthcare demands (6, 7).

Lumbar disc herniation is a major cause of low back pain, sciatica, and radicular leg pain, imposing a burden on individuals and society (8). The etiology of leg pain (sciatica) from lumbar disc herniation primarily involves mechanical compression of the nerve root (9).

Traditional treatments include medications (e.g., nonsteroidal anti-inflammatory drugs and muscle relaxants) and physical therapy. In some cases, surgical intervention may be needed. However, these traditional therapies have certain limitations, such as the side effects of the medication and the risks of surgery. Therefore, it is important to find safe and effective alternative therapies. Acupuncture is an important part of traditional Chinese medicine (TCM) that aims to treat diseases by stimulating specific acupuncture points to regulate the body’s physiological functions. In recent years, acupuncture has shown potential in the treatment of various painful conditions, including sciatica (10–12). Acupuncture can relieve pain through a variety of mechanisms, such as stimulating the endogenous analgesic system, releasing substances such as endorphins, regulating neuroinflammatory responses and attenuating inflammation in nerve roots (13).

There have been several systematic reviews and meta-analyses of the use of acupuncture for the treatment of sciatica (14–16). However, the available evidence remains controversial due to the quality of the included studies, the heterogeneity of interventions, and the length of follow-up (17). Therefore, it is necessary to conduct a high-quality meta-analysis. We focused on the literature published in the past decade to systematically evaluate the latest clinical evidence of acupuncture treatment for sciatica caused by lumbar intervertebral disc herniation.

This study aims to critically evaluate the efficacy of acupuncture in the treatment of sciatica caused by lumbar disc herniation through a systematic review and meta-analysis. This study will provide clinicians with a more reliable evidence-based basis and provide a reference for future research directions.

## 2 Methods

### 2.1 Protocol and guidance

The protocol of this study was registered on the International Prospective Register of Systematic Reviews (PROSPERO), and the registration number was CRD420251067853. This systematic review followed the guidelines of the Preferred Reporting Items for Systematic Reviews and Meta-analyses (PRISMA) (18).

### 2.2 Search strategy

To ensure the quality of the literature, we systematically searched English electronic databases to identify all relevant randomized controlled trials (PubMed, Web of Science, Embase, and the Cochrane Library). The search range ranged from the last decade (2015.05–2025.05). The search was conducted via MeSH vocabulary combinations and the following group terms: (1) acupuncture (acupuncture, electroacupuncture, needle, needling, etc.), (2) sciatica (sciatic nerve pain, sciatic neuralgia, sciatic neuropathy, sciatic pain, etc.), and (3) herniated disk (herniated disc, intervertebral disc herniation, disc herniation, etc.) The detailed search strategies for each database are presented in Supplementary Table 1.

### 2.3 Study selection: inclusion and exclusion criteria

#### 2.3.1 Inclusion criteria

Patients: Adults aged 18 years and older were diagnosed with sciatica due to a herniated disk, regardless of sex, ethnicity, or socioeconomic status.

Interventions: The experimental group received acupuncture treatment, which may include various techniques (e.g., manual acupuncture, electroacupuncture).

Comparisons: The control group received either sham acupuncture, no treatment, or standard care (e.g., physical therapy, medication, conventional acupuncture) for sciatica.

Outcomes: At least one of the following outcomes was reported: pain intensity, functional improvement, and quality of life.

Study Design: Randomized controlled trials (RCTs) and quasi-experimental studies.

#### 2.3.2 Exclusion criteria

Patients: Studies involving participants under 18 years of age or those not diagnosed with sciatica due to the herniated intervertebral disc.

Interventions: Studies that do not specify the type of acupuncture intervention, number of sessions, duration of each session, or total treatment duration.

Comparisons: Studies that did not include a control group receiving sham acupuncture, no treatment, or other standard care.

Outcomes: Studies that do not report on at least one of the specified outcomes.

Study Design: Nonrandomized controlled trials, observational studies, animal studies, case reports, reviews, meta-analyses, or duplicates.

**Figure 1:**
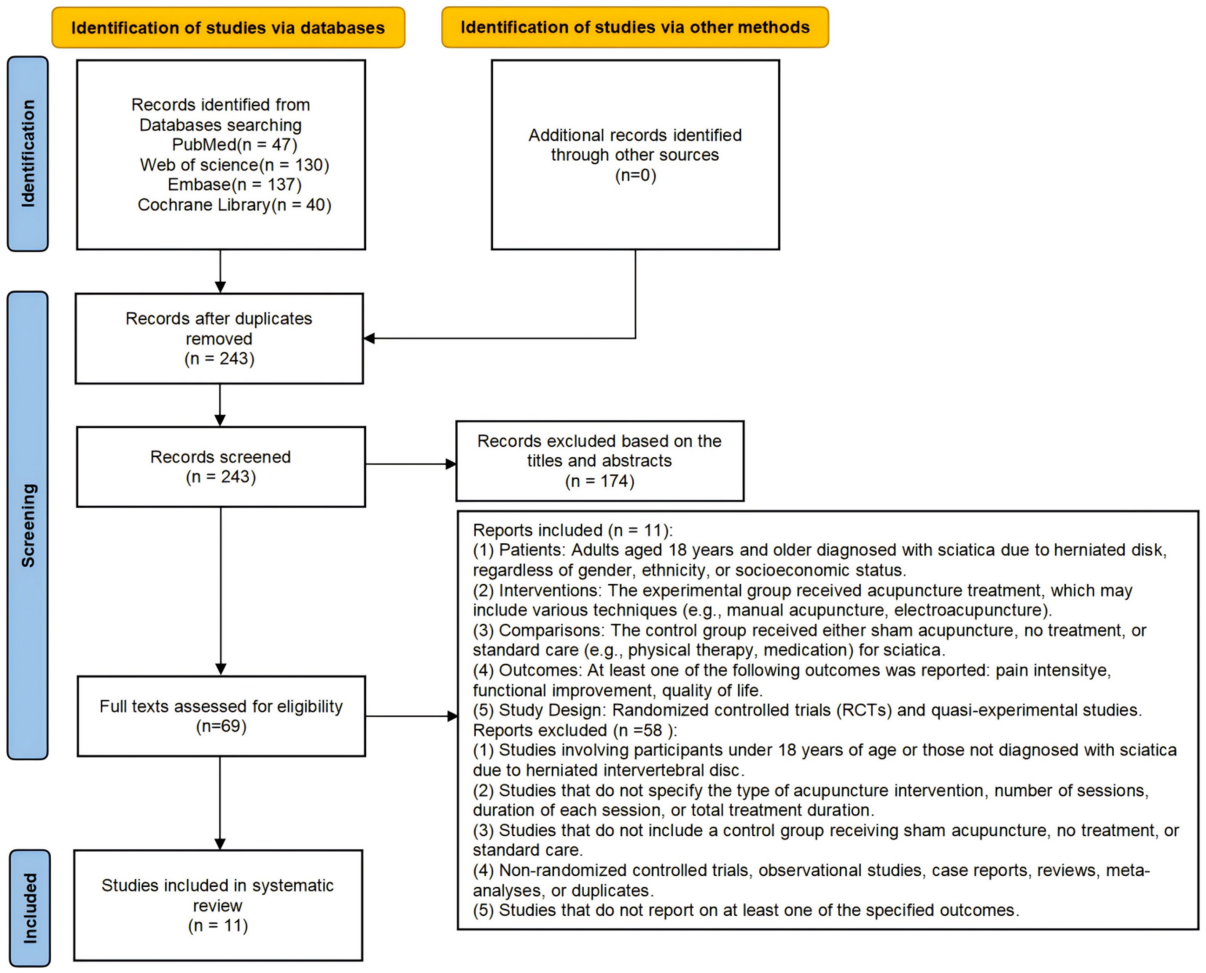
The systematic review and meta-analysis (PRISMA) flowchart of study selection.

This selection process is shown in Figure 1.

### 2.4 Study selection and data extraction

The retrieved records were imported into Endnote, and duplicates were removed. Using a standardized data extraction form, two reviewers (Zhen Qu and Jiangyi Ju) independently extracted detailed information about the included studies, including investigator name, year of publication, study design, sample size, patient characteristics, interventions (e.g., acupuncture points, duration of treatment, frequency), control group interventions, outcome measures, and adverse effects. Discrepancies between reviewers were resolved through discussion with a third independent reviewer (LiHua Peng). If study data are missing or incomplete, an attempt will be made to contact the authors. Any disputes arising during the data extraction process will also be resolved through discussion or consultation.

### 2.5 Risk of bias assessment

Two reviewers (Zhen Qu and Jiangyi Ju) independently assessed the risk of bias for each included study. The methodological quality of the included studies was assessed via the Cochrane Risk of Bias 2.0 (RoB 2.0) tool (19). Assessments included bias arising from the randomization process, bias due to deviations from intended interventions, bias due to missing outcome data, bias in measurement of the outcome, and bias in selection of the reported result. The studies were classified into three levels: low risk, some concern, and high risk.

### 2.6 Statistical analysis

Data analyses were conducted via Stata (version 12.0) and R (version 4.5.1) software. Continuous outcomes such as pain intensity visual analog scale (VAS) scores, the functional disability Oswestry Disability Index (ODI), and the mean difference (MD) or standardized mean difference (SMD) were used as the effect measures, each with a 95% confidence interval (95% CI). For dichotomous outcomes, such as the total effective rate, the relative risk (RR) or odds ratio (OR) was employed as the effect measure, as was the 95% CI. Heterogeneity between studies was assessed via the Cochran’s Q statistic and the I^2^ statistic (20). If I^2^ ≤ 50% and the *p* value >0.10, heterogeneity was considered low, and a fixed-effects model was used for the analysis. If I^2^ > 50% or the *p* value ≤0.10, significant heterogeneity was deemed present, and a random effects model was employed (20). Subgroup analyses and meta-regression were performed to explore the potential influence of categorical and continuous variables on the results (21). Publication bias was evaluated qualitatively by visually inspecting funnel plot asymmetry. Given the potential for publication bias, a sensitivity analysis was further conducted via leave-one-out analysis.

### 2.7 Subgroup analysis

We performed a subgroup analysis on the basis of the types of control interventions (sham acupuncture, other types of acupuncture, standard care).

## 3 Results

### 3.1 Search results

The flow diagram of the screening process is shown in Figure 1. A total of 354 records were retrieved from the search. After removing duplicates, 243 records were screened for potential relevance on the basis of their titles and abstracts. Among these, 174 records were excluded, leaving 69 records for full-text evaluation. Through the screening process, we ultimately included 11 studies (22–32). After full-text assessment, 58 studies were excluded. The primary reason for exclusion was that the interventions in these studies did not meet the inclusion criteria. Additionally, we excluded animal studies, reviews, meta-analyses, case reports and observational studies because they were not randomized controlled trials (RCTs).

This review included 11 studies involving a total of 868 participants. Almost all the studies were conducted in China, with 3 studies (24, 30, 31) being Chinese publications and the remainder being English publications. All studies were randomized controlled trials (RCTs), with one being a multi-center trial (26). The sample sizes of the included studies ranged from 30 to 216 participants. All participants in the included trials were required to have a diagnosis of sciatica. All studies reported pain intensity via the VAS score for leg pain, and 7 studies reported the ODI score (22, 23, 25, 26, 28, 30, 31). All intervention groups received acupuncture, including acupuncture (22, 25, 26, 28), electroacupuncture (23), and specific acupuncture (24, 27, 29–32). Among the control groups, 4 studies (22, 25, 26, 28) used sham acupuncture, 2 studies (23, 29) used standard care (medication, electrotherapy), and 5 studies (24, 27, 30–32) used conventional acupuncture. Further details of these studies are summarized in Table 1.

**Table 1:** Characteristics of the included studies.

Author	Publication year	Sample Size		Age, mean (SD), year		Interventions		Outcomes
		Acupuncture group	Control group	Acupuncture group	Control group	Acupuncture group	Control group	
Li C (22)	2021	37	36	56.9 (8.3)	57.3 (9.7)	Acupuncture	Sham acupuncture	VAS, ODI
Zhang X (23)	2017	50	50	54.3 (12.4)	51.1 (13.0)	Electroacupuncture	Medium-frequency electrotherapy	NRS, ODI
Qiu L (24)	2016	30	30	43.1 (15.3)	43.2 (14.3)	Sciatic nerve and routine acupuncture	simple routine acupuncture	VAS, PRI
Wei XY (25)	2024	25	25	60.5 (11.3)	54.3 (13.2)	Acupuncture	Sham acupuncture	VAS, ODI, SFBI
Tu JF (26)	2024	108	108	51.6 (14.9)	50.9 (15.5)	Acupuncture	Sham acupuncture	VAS, ODI, SFBI, SF-36
Li C (27)	2023	50	50	50.4 (6.9)	50.2 (6.9)	Canggui Tanxue technique acupuncture	Huantiao point acupuncture	VAS, JOA, RDQ
Huang ZL (28)	2019	23	23	63.0 (14.0)	63.0 (11.0)	Acupuncture	Sham acupuncture	VAS, ODI
Gyeltshen D (29)	2025	35	35	41.4 (12.8)	41.1 (13.1)	Warm acupuncture	Gabapentin	VAS, RDQ
Pan HT (30)	2022	38	38	57.0 (10.0)	55.0 (10.0)	Fanzhen Jieci acupuncture	Conventional acupuncture	VAS, ODI
Zai FL (31)	2018	30	30	55.1 (5.4)	54.6 (5.4)	Warming needle moxibustion acupuncture	Conventional acupuncture	VAS, ODI
Liu CH (32)	2019	15	15	63.2 (8.2)	54.3 (14.9)	“High-dose” manual acupuncture	“Low-dose” manual acupuncture	VAS, RDQ

### 3.2 Risk of bias assessment

**Figure 2:**
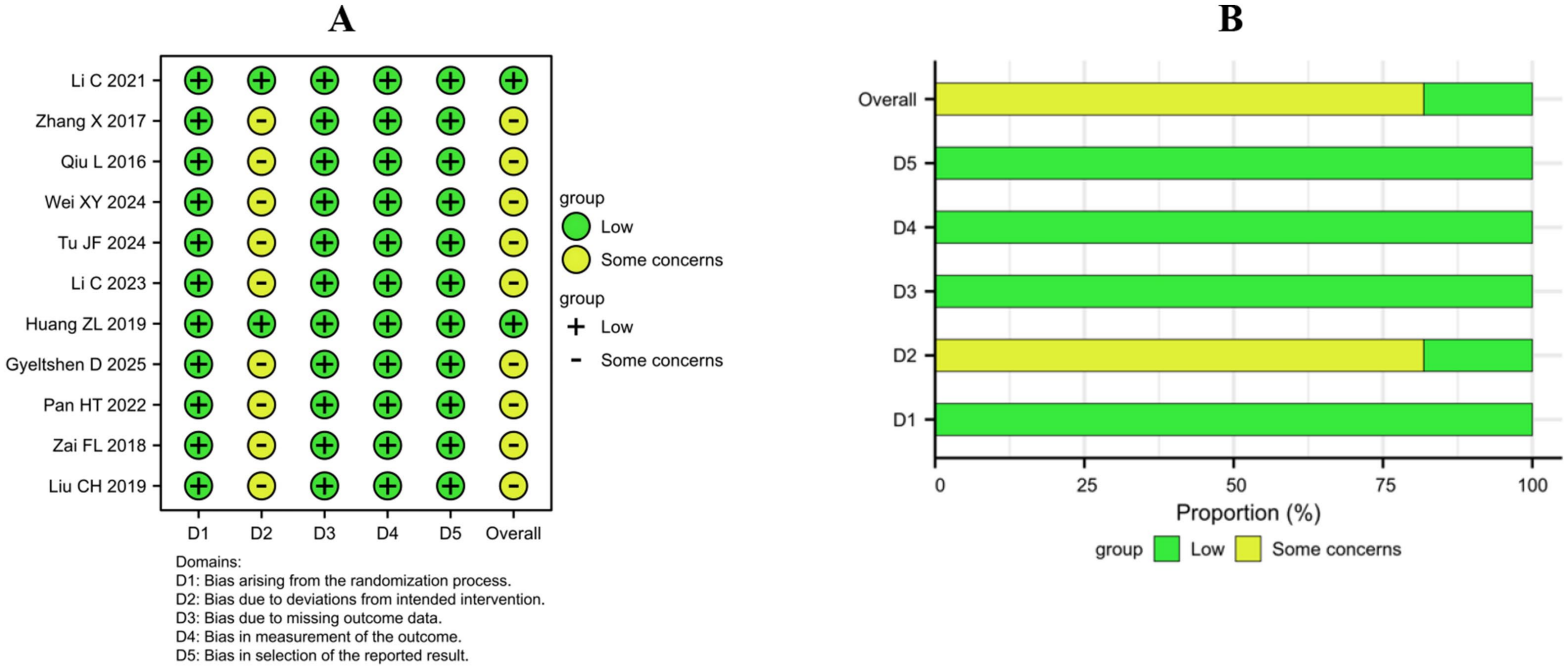
Risk of bias within the included studies. **(A)** Risk of bias summary. **(B)** Risk of bias graph.

The assessment results of “risk of bias” in each domain for individual studies are shown in Figure 2A, while the percentage results of risk assessment for each domain are presented in Figure 2B. This study employed the Cochrane risk of bias 2.0 (RoB 2.0) tool to evaluate the methodological quality of the 11 included studies. Overall, 2 studies (22, 28) had a low risk of bias, 9 studies (23–27, 29–32) had a potential risk of bias, and none had a high risk of bias.

All 11 studies used a random allocation order, which is considered to have a low risk of bias. In terms of intervention measures, the main problem is the risk of bias caused by the nonblind method of carriers and people delivering the interventions, which is related to the characteristics of acupuncture. In the process of acupuncture treatment, it is difficult to implement blind acupuncture. However, 2 studies (22, 28) were judged to have a low risk of bias because they used adhesive pads to ensure blinding during sham acupuncture. Almost all 11 studies reported complete intervention outcomes for randomized participants, which were considered to have a low risk of bias. All 11 studies reported VAS scores as the primary outcome for assessing pain intensity, which is considered a low risk of bias. All 11 studies analyzed their data in accordance with a prespecified analysis plan that was finalized before unblinded outcome data were available for analysis, which was considered to have a low risk of bias.

### 3.3 Effect of interventions

#### 3.3.1 VAS (visual analog scale) score for leg pain

Previous studies (33, 34) have used the total effective rate as the primary outcome measure, but among the 11 studies included in this research, not all reported the number of effective cases treated with acupuncture. Therefore, we selected the VAS score (0–10 cm scale (22, 25, 26, 28, 30) and 0–100 cm scale (23, 24, 27, 29, 31, 32), with 0 cm representing no pain and 10 cm or 100 cm representing extreme pain) at 4 weeks postintervention as the primary outcome to evaluate the effectiveness of acupuncture for sciatica.

**Figure 3:**
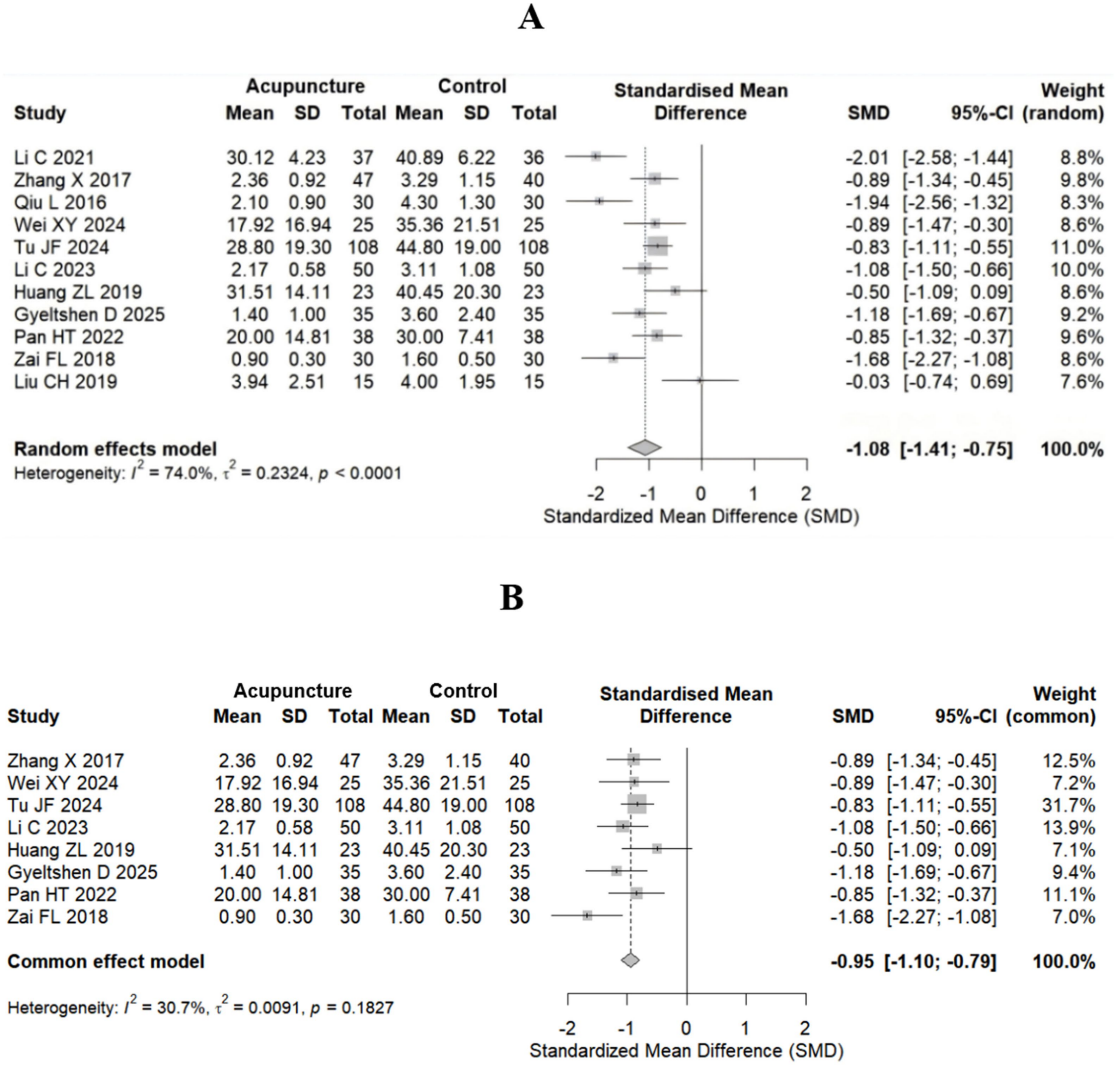
Forest plot for the VAS score. **(A)** All studies included (11 studies). **(B)** After highly heterogeneous studies (8 studies) were excluded.

This study included 11 randomized controlled trials (RCTs) with an overall sample size of 868 patients. A random-effects model was used to pool the standardized mean differences (SMDs), revealing that acupuncture treatment for sciatica demonstrated significant clinical efficacy (SMD = −1.08, 95% CI: −1.41–−0.75) (Figure 3A). This effect size exceeded Cohen’s d threshold for a large effect (d = 0.8) (34), indicating superior pain relief in the acupuncture group compared with the control group. Among the 11 studies, 9 studies (22–27, 29–31) reported statistically significant benefits (95% CI not 0), whereas Liu CH (32) reported no significant difference, potentially attributable to its smaller sample size (*n* = 15 in each group).

There was high heterogeneity among the studies (I^2^ = 74.0%). Therefore, after highly heterogeneous studies (22, 24, 32) were excluded, 8 studies (total sample size *n* = 718) were ultimately included, and a fixed-effect model combined with the standardized mean difference (SMD) was used. The results revealed that the efficacy of acupuncture in the treatment of sciatica was significantly better than that in the control group, with an SMD of −0.95 (95% CI: −1.10–−0.79) (Figure 3B). The effect size still exceeded Cohen’s d threshold for large effects (d = 0.8) (34). Heterogeneity was significantly reduced to an acceptable level (I^2^ = 30.7%, *p* = 0.1827), supporting the use of a fixed-effects model. The effectiveness of acupuncture on sciatica is more stable.

**Figure 4:**
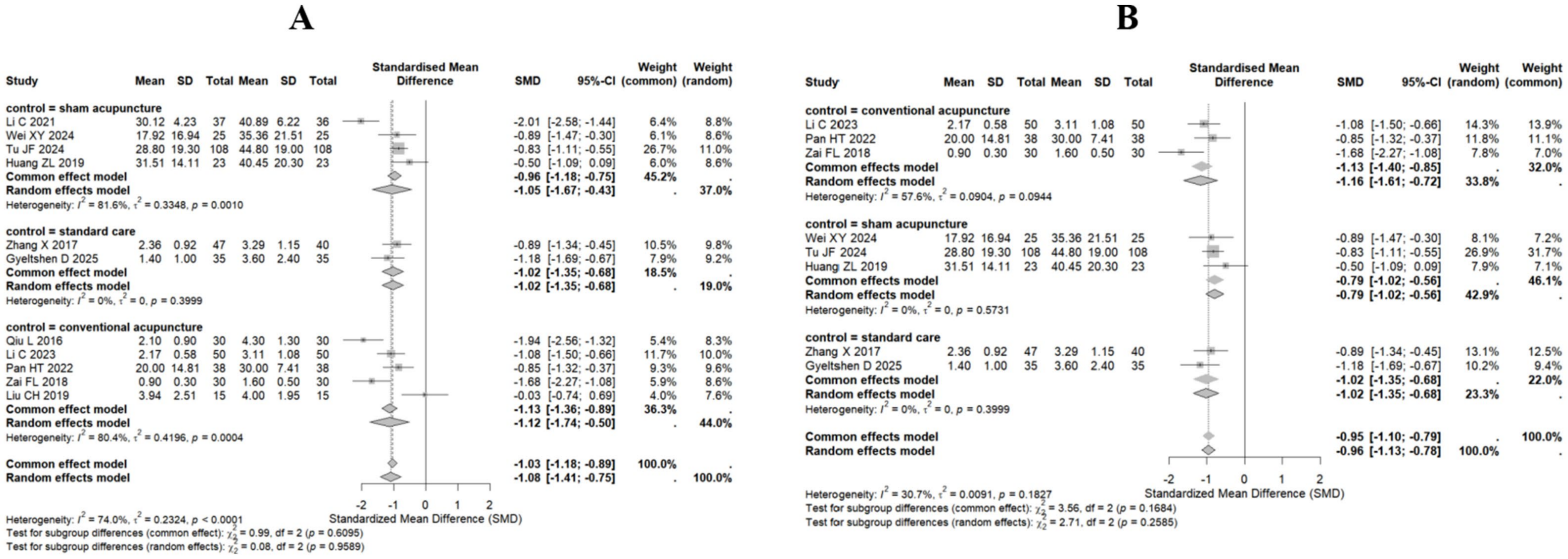
Forest plot for subgroup analysis of the VAS score. **(A)** All studies included (11 studies). **(B)** After highly heterogeneous studies (8 studies) were excluded.

We also performed subgroup analysis on the basis of the types of control interventions. The subgroup analysis indicated that acupuncture treatment for sciatica demonstrated significant efficacy across different control types: the sham acupuncture control group (22, 25, 26, 28) presented a pooled standardized mean difference (SMD = −1.05 95% CI: −1.67 to −0.43); the standard care control group (23, 29) presented homogeneous significant efficacy (SMD = −1.02, 95% CI: −1.35 to −0.68; I^2^ = 0%, *p* = 0.3999); and the conventional acupuncture control group (24, 27, 30–32) presented a pooled effect size of SMD = −1.12 (95% CI: −1.74 to −0.50). All studies except Liu CH (32) (SMD = −0.03, 95% CI: −0.74 to 0.69) presented significant effectiveness. The test for subgroup differences revealed no statistically significant differences in either the fixed-effects model (*p* = 0.6095) or the random-effects model (*p* = 0.9589), indicating that acupuncture efficacy is not influenced by the control type. The overall pooled effect (11 studies) maintained a large effect size (SMD = −1.03, 95% CI: −1.18–−0.89) (Figure 4A).

Subgroup analyses also revealed substantial heterogeneity. Consequently, following the exclusion of highly heterogeneous studies (22, 24, 32), both fixed-effect and random-effects models were employed, utilizing the standardized mean difference (SMD) for pooled analysis. Results demonstrated consistent efficacy across all control groups (conventional acupuncture, sham acupuncture, and standard care). Under the random-effects model, the conventional acupuncture group exhibited an SMD of −1.16 (95% CI: −1.61 to −0.72), indicating moderate heterogeneity (I^2^ = 57.6%). The sham acupuncture group yielded an SMD of −0.79 (95% CI: −1.02 to −0.56), with no observed heterogeneity (I^2^ = 0%). The standard care group demonstrated an SMD of −1.02 (95% CI: −1.35 to −0.68), also without heterogeneity (I^2^ = 0%). The overall pooled SMD under the random-effects model was −0.96 (95% CI: −1.13 to −0.78), with total heterogeneity quantified at 30.7%. Subgroup differences were statistically non-significant (*p* = 0.2585), further indicating consistent treatment effects across control groups (Figure 4B).

The meta-regression analysis was performed using a mixed-effects model (Supplementary Figure 1). The results indicated the presence of residual heterogeneity (I^2^ = 9.43%), with the model explaining 31.28% of the heterogeneity. This suggests that the included moderating variable (type of control intervention) partially explains variation between studies, although the overall effect remained statistically non-significant. When sham acupuncture and standard care served as control groups, their influence on the intervention effect size exhibited no significant difference compared to the conventional acupuncture group (*p* > 0.05), indicating that the type of control intervention did not substantially modify the intervention’s efficacy. Across all study designs, the intervention’s effect size remained consistent irrespective of the control intervention type, demonstrating robust stability in the intervention’s effectiveness.

**Figure 5:**
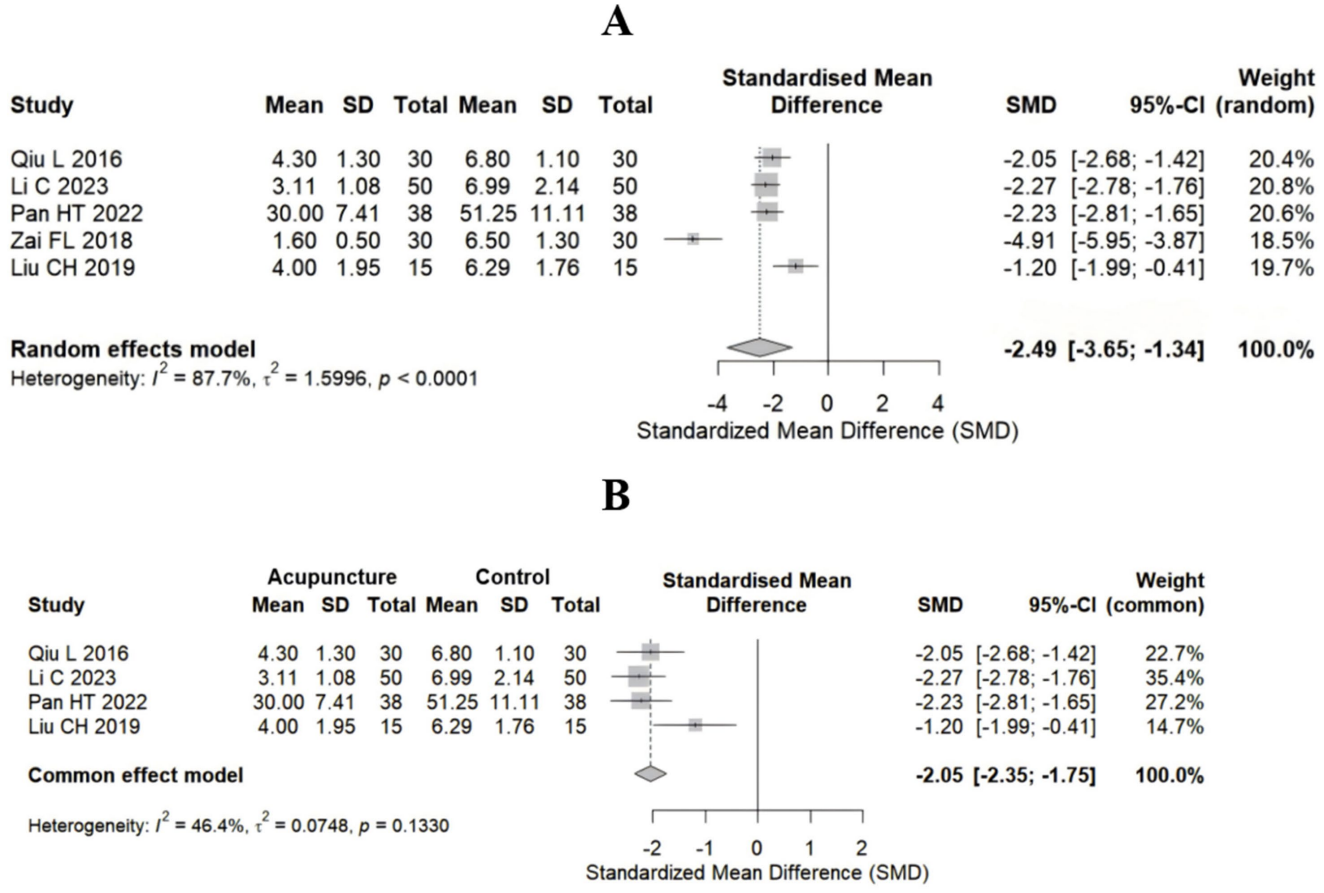
Forest plot for the VAS score in the studies that used acupuncture as control group. **(A)** Control group vs. baseline group (5 studies); **(B)** after the exclusion of highly heterogeneous studies (4 studies).

Among the 11 studies, 5 studies (24, 27, 30–32) used acupuncture as the control treatment. To demonstrate the efficacy of acupuncture for sciatica, we extracted the VAS score at 4 weeks postintervention and baseline from these 5 studies and pooled the effect sizes. The random-effects model results revealed that all studies confirmed significant clinical benefits of acupuncture treatment compared with baseline levels, with a pooled SMD of −2.49 (95% CI: −3.65–−1.34), far exceeding Cohen’s large effect threshold (SMD > 0.8) (Figure 5A). The extreme effect size observed in (31) may be the main reason for the high heterogeneity (I^2^ = 87.7%). After excluding study (31) that may have led to high heterogeneity, a fixed-effects model was used to repool the effect sizes. The results similarly demonstrated the effectiveness of acupuncture for sciatica (Figure 5B).

#### 3.3.2 ODI (Oswestry Disability Index)

**Figure 6:**
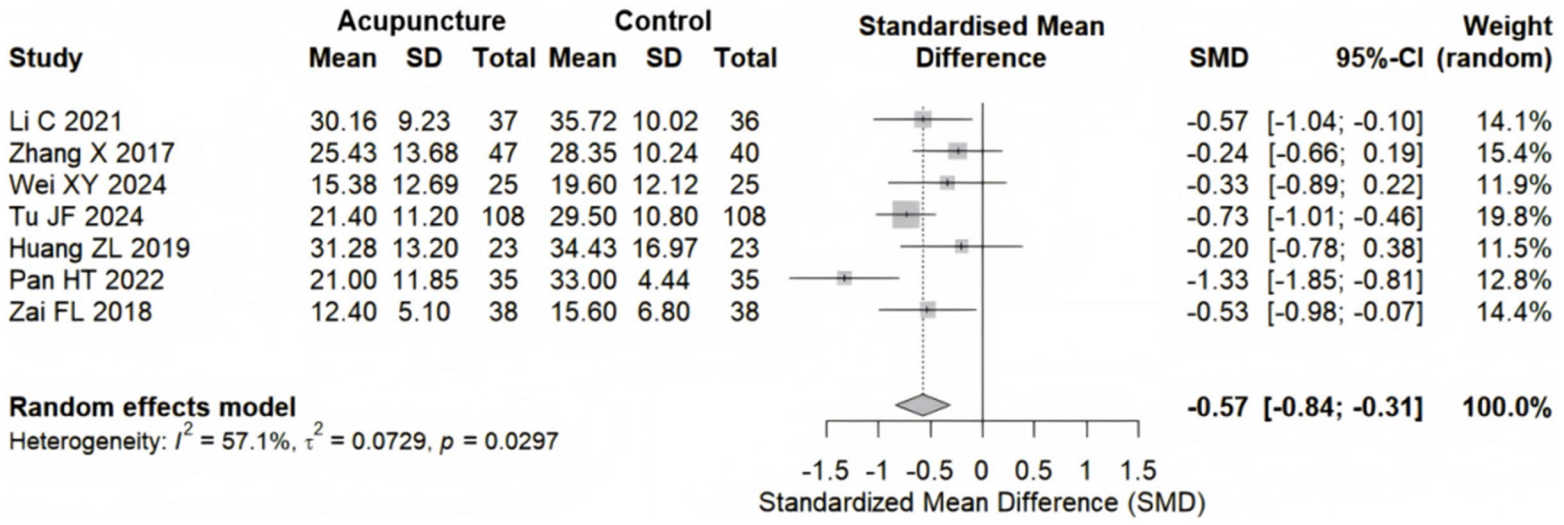
Forest plot for the ODI.

7 studies (22, 23, 25, 26, 28, 30, 31) with 621 participants examined the efficacy of acupuncture on the ODI. Pooled results demonstrated that acupuncture significantly outperformed the control group in improving the ODI for sciatica patients, with a random effects model yielding an SMD of −0.57 (95% CI: −0.84 to −0.31), reaching Cohen’s d threshold for a medium effect (d = 0.5) (34). Moderate heterogeneity was observed among the studies (I^2^ = 57.1%, *p* = 0.0297) (Figure 6).

### 3.4 Sensitivity analysis

**Figure 7:**
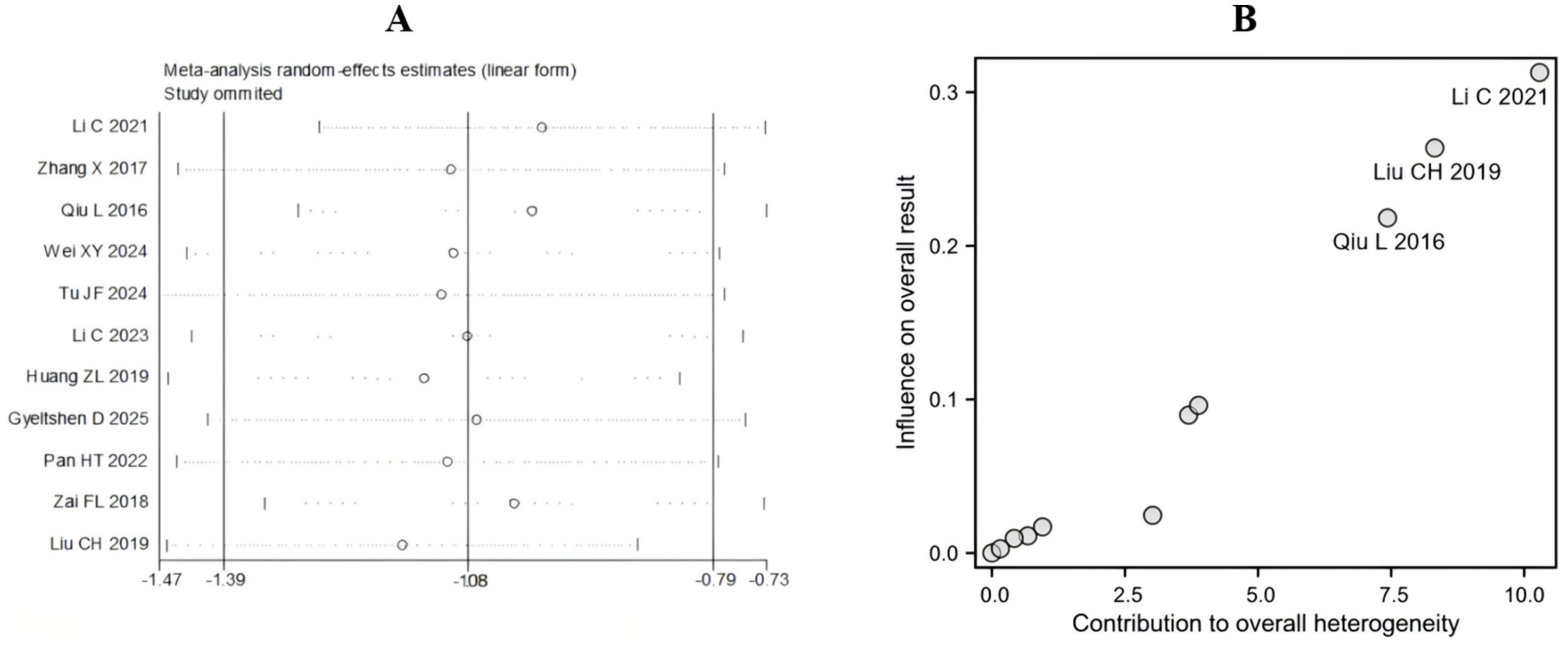
Sensitivity analysis. **(A)** Leave-one-out method for VAS. **(B)** Baujat plot for VAS.

With respect to the high heterogeneity found in the effect of acupuncture on the VAS pain score (I^2^ = 74%), we performed a sensitivity analysis. This sensitivity analysis employed the leave-one-out method, confirming the robustness of the meta-analysis results for acupuncture treatment of sciatica (Figure 7A). When any one of the 11 included studies was sequentially removed, the pooled standardized mean difference (SMD) under the random-effects model fluctuated between −1.47 and −0.73 (original pooled SMD = −1.08), with all adjusted effect sizes remaining statistically significant (Supplementary Table 2). Moreover, Baujat plot analysis identified three studies (22, 24, 32) exerting disproportionate influence on both heterogeneity and the pooled VAS score estimates (Figure 7B). These results demonstrated that the heterogeneity primarily originated from individual extreme studies, but the core conclusion (acupuncture significantly remits sciatica) was highly robust.

### 3.5 Publication bias

**Figure 8:**
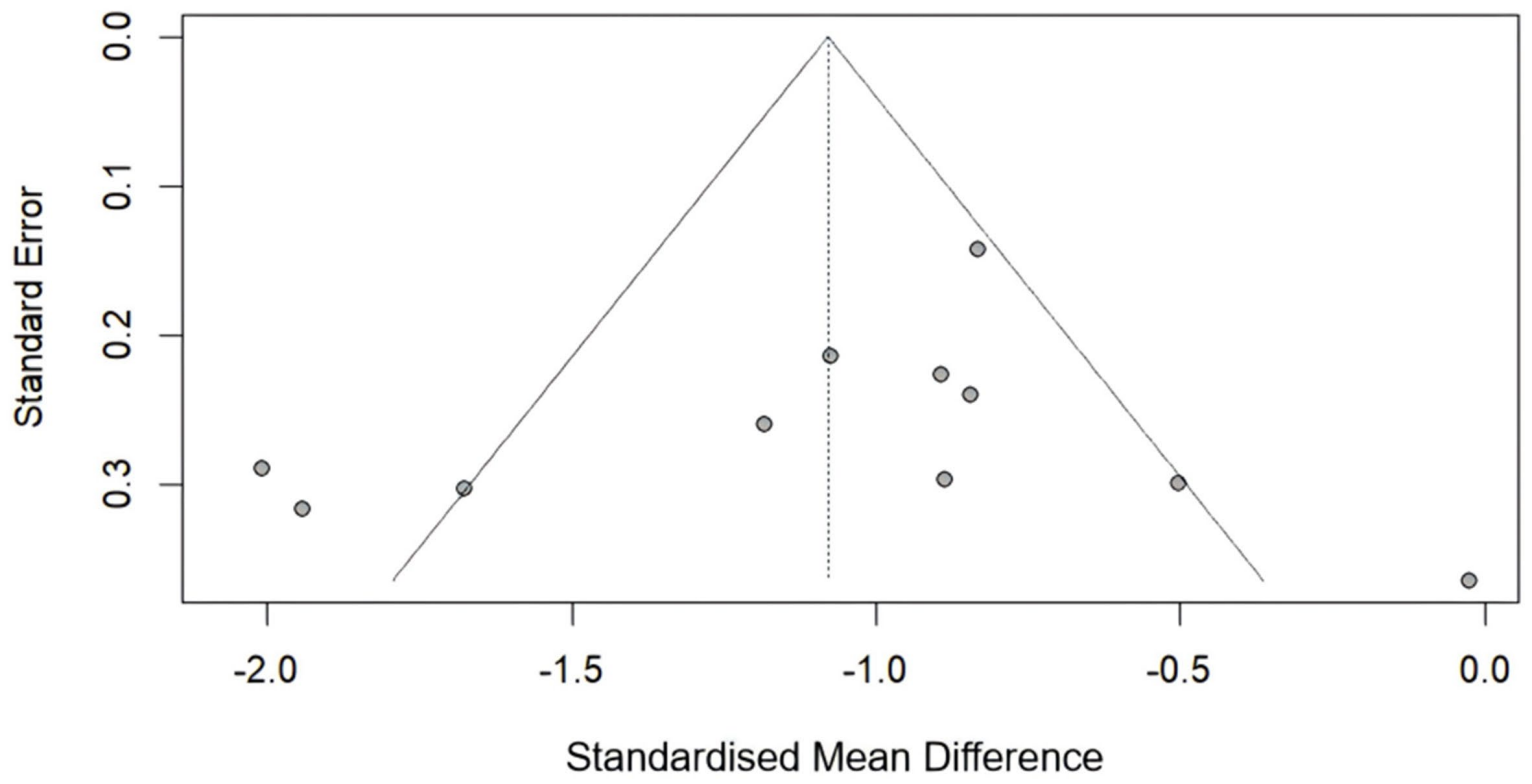
Funnel plot for the VAS score.

We generated a funnel plot (Figure 8) and used Eggee’s test and Begg’s test (Supplementary Table 3) to calculate the VAS scores. The symmetrical funnel distribution and the results of Egger’s test (*p* = 0.4755) and Begg’s test (*p* = 0.6971) support the absence of significant publication bias in this study.

## 4 Discussion

This systematic review and meta-analysis revealed that acupuncture significantly reduced the VAS score and the ODI. Crucially, the subgroup analysis revealed no significant difference in efficacy between the types of control (sham acupuncture, standard care or traditional acupuncture), which strengthens the specific therapeutic effect of acupuncture beyond the placebo effect. Despite moderate to high statistical heterogeneity (I^2^ = 57.1% for ODI outcomes; 74.0% for overall pain scores), the directional consistency of effects (SMD: −0.84 to −0.31 for ODI; −1.41 to −0.75 for VAS score) robustly supports the efficacy of acupuncture in alleviating sciatica. This heterogeneity likely stems from methodological diversity (e.g., variations in acupuncture protocols (e.g., electroacupuncture and manual acupuncture), outcome measurement tools (VAS and ODI), and control interventions (sham acupuncture and standard care)). Such clinical diversity is inherent in traditional medicine meta-analyses and does not invalidate efficacy conclusions when effects remain consistently favorable (35).

The symmetrical funnel distribution (Egger’s *p* = 0.475; Begg’s *p* = 0.69) indicates a low risk of publication bias, despite isolated outliers [e.g., (32)]. This suggests that small studies with null or negative findings were adequately captured, strengthening confidence in the pooled effect estimates.

The moderate improvement in the VAS score following acupuncture aligns with conventional therapies such as NSAIDs (36) but has superior safety. Future trials should standardize acupuncture protocols (e.g., using STRICTA guidelines) and prioritize patient-centered outcomes (e.g., duration of pain recovery and functional recovery) to bridge evidence-practice gaps.

Acupuncture, as a therapeutic approach for sciatica, has been extensively studied, particularly in the treatment of sciatica caused by herniated discs (37). Multiple studies have demonstrated that acupuncture can effectively alleviate sciatic pain and improve patients’ quality of life (38, 39). A systematic review and meta-analysis revealed that acupuncture is more effective than medication in treating sciatica and can enhance drug efficacy (14). Furthermore, the safety profile of acupuncture has been confirmed, with fewer adverse reactions, making it a viable alternative for patients who avoid or cannot tolerate medication (15).

## 5 Limitations

While the therapeutic efficacy of acupuncture has been preliminarily confirmed, existing research still faces several limitations. (1) Significant heterogeneity exists in acupuncture protocols across studies, including variations in acupoint selection, needling techniques, and treatment frequency, making it challenging to establish optimal treatment regimens. Future research should focus on developing standardized acupuncture protocols to better evaluate their effectiveness and facilitate broader application. (2) Current studies generally have small sample sizes and short follow-up periods, limiting long-term efficacy assessment. Therefore, future research requires larger-scale, well-designed randomized controlled trials to further validate the therapeutic outcomes and durability of acupuncture.

## 6 Conclusion

Acupuncture has statistically and clinically significant benefits for herniated disc-related sciatica. While methodological limitations in the extant literature necessitate cautious interpretation, these findings support the inclusion of acupuncture in multidisciplinary pain management. Future high-quality RCTs addressing current methodological shortcomings will strengthen evidence-based implementation.

## Data Availability

The original contributions presented in the study are included in the article/Supplementary material, further inquiries can be directed to the corresponding author.
