# Supplementary material for: Effectiveness of acupuncture in the treatment of chronic sciatica from herniated disks: a systematic review and meta-analysis
Source: Front Med (Lausanne). 2026 Jan 22;13:1689124. doi: 10.3389/fmed.2026.1689124 (PMC12872920; doi:10.3389/fmed.2026.1689124)
Supplement: Supplementary file 1 [file Table_1.docx]

**Supplementary Table 1** Literature search strategy for this meta-analysis.

Table 1-1. Search strategy for Pubmed (47)

| **Number** | **Search terms** |
| --- | --- |
| #1 | Acupuncture[MeSH Terms] OR Acupuncture[Title/Abstract] OR Acupuncture Therapy[Title/Abstract] OR Acupuncture Analgesia[Title/Abstract] OR Acupuncture, Electron[Title/Abstract] OR Electroacupuncture[Title/Abstract] OR Needle[Title/Abstract] OR Needling[Title/Abstract] |
| #2 | Sciatica[MeSH Terms] OR Sciatica[Title/Abstract] OR Sciatic Nerve Pain[Title/Abstract] OR Sciatic Pain[Title/Abstract] OR Sciatic Neuralgia[Title/Abstract] OR Sciatic Neuropathy[Title/Abstract] |
| #3 | Herniated Disk[MeSH Terms] OR Herniated Disk[Title/Abstract] OR Herniated Disc[Title/Abstract] OR Intervertebral Disc Herniation[Title/Abstract] OR Disc Herniation[Title/Abstract] |
| #4 | #1 AND #2 AND #3 |

Table 1-2. Search strategy for Web of Science (130)

| **Number** | **Search terms** |
| --- | --- |
| #1 | TS=((Acupuncture) OR (Acupuncture Therapy) OR (Acupuncture Analgesia) OR (Acupuncture, Electron) OR (Electroacupuncture) OR (Needle)) |
| #2 | TS=((Sciatica) OR (Sciatic Nerve Pain) OR (Sciatic Pain) OR (Sciatic Neuralgia) OR (Sciatic Neuropathy)) |
| #3 | TS=((Intervertebral Disc Displacement) OR (Protruded Disc) OR (Protruded Disk) OR (Intervertebral Disk Displacement) OR (Herniated Disk) OR (Slipped Disk) OR (Disk Prolapse) OR (Prolapsed Disk) OR (Herniated Disc) OR (Herniated Disk) OR (Slipped Disc) OR (Prolapsed Disc) OR (Disc Herniation) OR (Intervertebral Disc Herniation) OR (Intervertebral Disk Herniation) OR (Disk Herniation) OR (Intervertebral Disk Protrusion) OR (Intervertebral Disc Protrusion) OR (Disc Protrusion) OR (Disk Protrusion)) |
| #4 | #1 AND #2 AND #3 |

Table 1-3. Search strategy for Embase (137)

| **Number** | **Search terms** |
| --- | --- |
| #1 | 'acupuncture'/exp |
| #2 | ('acupuncture' OR 'acupuncture therapy' OR 'acupuncture analgesia' OR 'electroacupuncture' OR 'acupuncture, electron' OR 'needle' OR 'needling'):ti,ab,kw |
| #3 | #1 OR #2 |
| #4 | 'sciatica'/exp |
| #5 | ('ischias' OR 'ischiatic neuralgia' OR 'ischiatic pain' OR 'pain, sciatic' OR 'sciatalgia' OR 'sciatic nerve pain' OR 'sciatic neuralgia' OR 'sciatic pain' OR 'sciatica' OR 'sciatic neuropathy'):ti,ab,kw |
| #6 | #4 OR #5 |
| #7 | 'intervertebral disk hernia'/exp |
| #8 | ('herniated disk' OR 'herniated disc' OR 'intervertebral disc herniation' OR 'disc herniation'):ti,ab,kw |
| #9 | #7 OR #8 |
| #10 | #3 AND #6 AND #9 |

Table 1-4. Search strategy for the Cochrane Library (40)

| **Number** | **Search terms** |
| --- | --- |
| #1 | MeSH descriptor: [Acupuncture] explode all trees |
| #2 | (Acupuncture OR “Acupuncture Therapy” OR “Acupuncture Analgesia” OR “Electroacupuncture” OR “Acupuncture, Electron” OR “Needle” OR “Needling”):ti,ab,kw |
| #3 | #1 OR #2 |
| #4 | MeSH descriptor: [Sciatica] explode all trees |
| #5 | (“Sciatica” OR “Sciatic Nerve Pain” OR “Sciatic Pain” OR “Sciatic Neuropathy” OR “Sciatic Neuralgia”):ti,ab,kw |
| #6 | #4 OR #5 |
| #7 | MeSH descriptor: [Intervertebral Disc Displacement] explode all trees |
| #8 | (“Herniated Disk” OR “Herniated Disc” OR “Disc Herniation” OR “Intervertebral Disc Herniation” OR “Disc Herniation”):ti,ab,kw |
| #9 | #7 OR #8 |
| #10 | #3 AND #6 AND #9 |

**Supplementary Figure 1** Result for meta regression


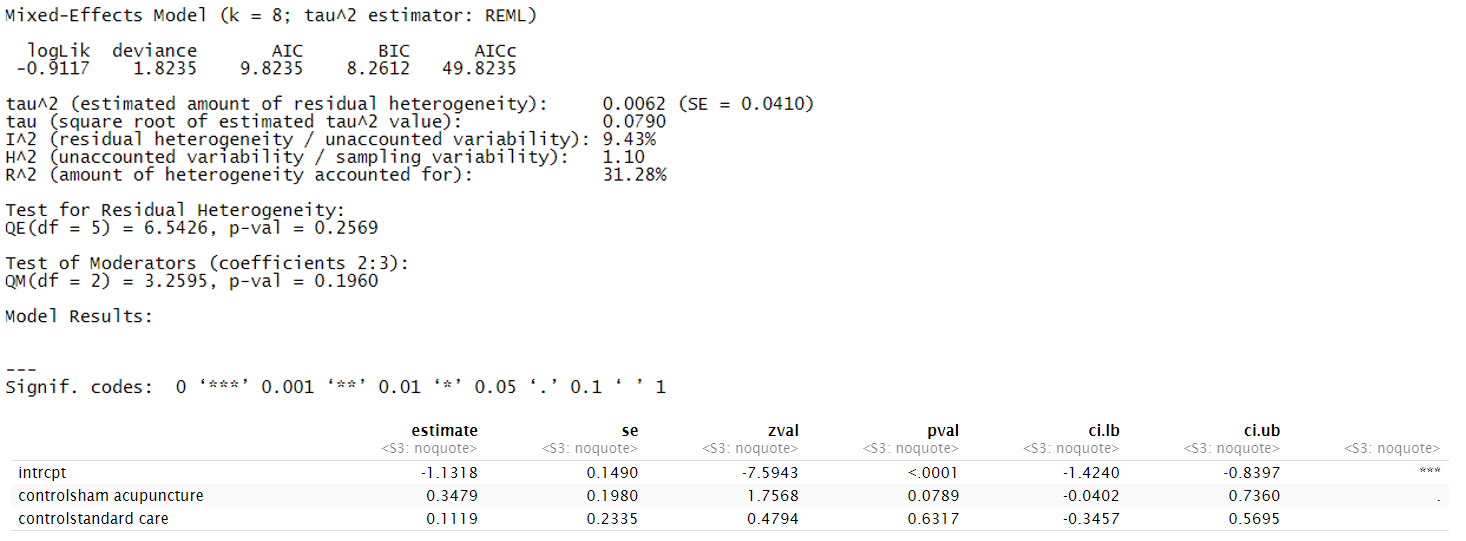


**Supplementary Table 2** Sensitivity analyses for outcomes

|  | SMD | 95%-CI | p-value | tau^2 | tau | I^2 | effect model |
| --- | --- | --- | --- | --- | --- | --- | --- |
| Omitting Li C 2021 | -0.9651 | [-1.1138; -0.7345] | < 0.0001 | 0.1516 | 0.3894 | 0.658 | Random |
| Omitting Zhang X 2017 | -1.0484 | [-1.2004; -0.8964] | < 0.0001 | 0.2696 | 0.5193 | 0.763 | Random |
| Omitting Qiu L 2016 | -0.9804 | [-1.1282; -0.8326] | < 0.0001 | 0.1800 | 0.4243 | 0.697 | Random |
| Omitting Wei XY 2024 | -1.0415 | [-1.1898; -0.8931] | < 0.0001 | 0.2661 | 0.5158 | 0.764 | Random |
| Omitting Tu JF 2024 | -1.1047 | [-1.4727; -0.9368] | < 0.0001 | 0.2672 | 0.5169 | 0.748 | Random |
| Omitting Li C 2023 | -1.0262 | [-1.1792; -0.8732] | < 0.0001 | 0.2758 | 0.5252 | 0.766 | Random |
| Omitting Huang ZL 2019 | -1.0657 | [-1.2140; -0.9174] | < 0.0001 | 0.2286 | 0.4781 | 0.744 | Random |
| Omitting Gyeltshen D 2025 | -1.0190 | [-1.1688; -0.8691] | < 0.0001 | 0.2715 | 0.5210 | 0.764 | Random |
| Omitting Pan HT 2022 | -1.0513 | [-1.2023; -0.9003] | < 0.0001 | 0.2659 | 0.5156 | 0.762 | Random |
| Omitting Zai FL 2018 | -0.9919 | [-1.1401; -0.8437] | < 0.0001 | 0.2249 | 0.4742 | 0.732 | Random |
| Omitting Liu CH 2019 | -1.0744 | [-1.2211; -0.9276] | < 0.0001 | 0.1666 | 0.4082 | 0.705 | Random |

**Supplementary Table 3** Result of publication bias

Egger’s test for VAS

**
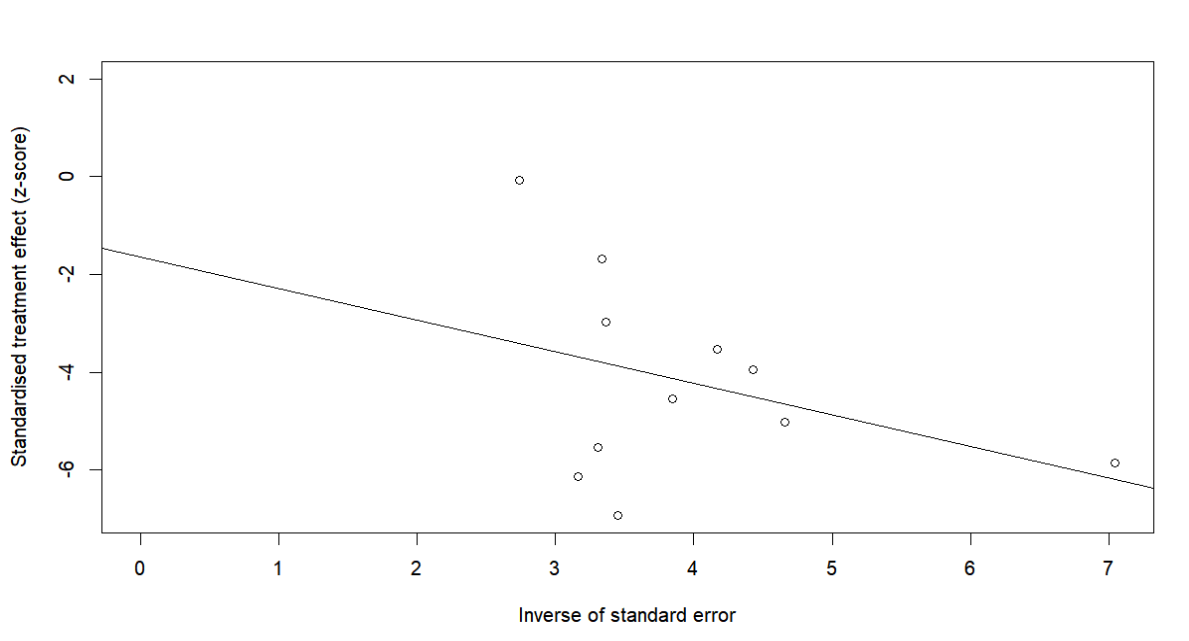
**

| Test result: t = -0.74, df = 9, p-value = 0.4755 |
| --- |
| Bias estimate: -1.6490 (SE = 2.2148) |
| Details: |
| - multiplicative residual heterogeneity variance (tau^2 = 4.0227) |
| - predictor: standard error |
| - weight: inverse variance |
| - reference: Egger et al. (1997), BMJ |

Begg’s test for VAS


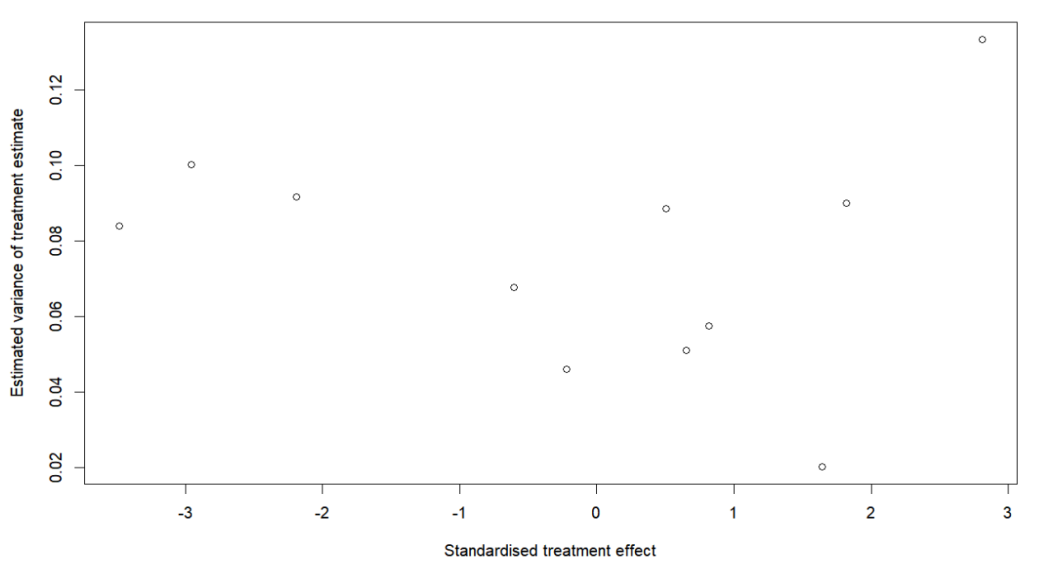


| Test result: z = -0.39, p-value = 0.6971 |
| --- |
| Bias estimate: -5.0000 (SE = 12.8452) |
|  |
| Reference: Begg & Mazumdar (1993), Biometrics |
